# Consequences of *β*-Thalassemia or Sickle Cell Disease for Ovarian Follicle Number and Morphology in Girls Who Had Ovarian Tissue Cryopreserved

**DOI:** 10.3389/fendo.2020.593718

**Published:** 2021-01-15

**Authors:** Linn Salto Mamsen, Stine Gry Kristensen, Susanne Elisabeth Pors, Jane Alrø Bøtkjær, Erik Ernst, Kirsten Tryde Macklon, Debra Gook, Ajay Kumar, Bhanu Kalra, Claus Yding Andersen

**Affiliations:** ^1^Laboratory of Reproductive Biology, The Juliane Marie Centre for Women, Children, and Reproduction, Copenhagen University Hospital, Rigshospitalet, Copenhagen, Denmark; ^2^Department of Obstetrics and Gynaecology, Regional Hospital Horsens, Horsens, Denmark; ^3^The Fertility Clinic, The Juliane Marie Centre for Women, Children, and Reproduction, Copenhagen University Hospital, Rigshospitalet, Copenhagen, Denmark; ^4^Reproductive Services and Melbourne IVF, Royal Women’s Hospital, Parkville, VIC, Australia; ^5^Department of Obstetrics and Gynaecology, University of Melbourne, Parkville, VIC, Australia; ^6^Ansh Labs LLC, Webster, TX, United States

**Keywords:** ovarian tissue cryopreservation, fertility preservation, thalassemia, sickle cell disease, genetic diseases

## Abstract

Women with *β*-thalassemia (BT) and sickle cell disease (SCD) have a high risk of infertility and premature ovarian insufficiency. Different fertility preserving strategies, including ovarian tissue cryopreservation (OTC) and oocyte cryopreservation has been considered, and healthy babies have been born after successful OTC and transplantation. We evaluated follicle number and follicle health in ovarian tissue from a cohort of BT and SCD patients who underwent OTC before the age of 18 years. Patients undergoing OTC from 2002 to 2019 were included. A total of 14 girls and adolescents with BT and four with SCD, aged 2.8–17.4 years at OTC were included together with a reference group of 43 girls and adolescents with non-anemia diseases considered to have normal ovaries aged 0.6–17.9 years at OTC. Ovarian follicle density was measured in cortex biopsies and compared to the reference group. Expression of proteins associated with follicular health was evaluated using immunohistochemistry. Follicles were detected in the ovarian cortex biopsies from all patients with BT and SCD. The follicle densities were within the 95% prediction interval of the reference group in all cases. A similar expression of six proteins essential for follicular health was detected using immunohistochemistry in BT, SCD, and references. OTC should be considered an option for young girls and adolescents with BT and SCD.

## Highlights

- Normal follicle density and morphology was detected in ovarian cortex biopsies in all young *β*-thalassemia or sickle cells disease patients.
- OTC should be considered before hematopoietic stem cell (HSC) transplantation.

## Introduction

Worldwide around 3–400,000 children are born annually with an inherited recessive hemoglobin disorder of which thalassemia and sickle cell disease (SCD) are the most prevalent (1, 2). Around 80% of these cases occur in low- and middle-income families in malaria-endemic regions stretching from sub-Saharan Africa and the Mediterranean to Southeast Asia (1, 3). The extremely high prevalence of thalassemia and SCD in regions with malaria or regions in which malaria previously was prevalent, reflects beneficial effects of these phenotypes. The malaria plasmodium parasite have a diminished survival and reproduction in the blood cells of patients with thalassemia and SCD resulting in better survival in these patients compared to subjects with normal red blood cells (4, 5). Moreover, there is a tradition for consanguineous marriage in some of these regions, which further increases the risk of thalassemia and SCD as well as carriers of these autosomal recessive disorders (3, 6).

*β*-thalassemia (BT) is a hemoglobin disorder caused by a deficiency in one or more *β*-globin genes causing abnormal hemoglobin formation and thereby reduced oxygen caring capacity of the erythrocytes (7). SCD is a multisystemic disorder characterized by anemia, increased hemolysis, and vaso-occlusive episodes (8, 9). Infertility in SCD is seen due to chronic inflammation, oxidative stress, transfusion-related hemochromatosis, reperfusion injury to the ovary and ischemia (10).

Transfusion-requiring BT (*i.e.* BT major) and SCD may lead to iron overload, resulting in liver damage, cardiac complications, endocrine dysfunctions, and compromised function of the reproductive organs (11–13). Transfusion related iron overload and suboptimal chelation therapy in childhood may lead to gonadal dysfunction including infertility, hypogonadotropic hypogonadism, delayed or absent sexual development, and osteoporosis, which are more common in TB than in SCD (11, 12, 14, 15). Excess iron may deposit in the endocrine organs causing oxidative stress and a dysfunction of the hypothalamic–pituitary–ovarian (HPO) axis leading to infertility in patients with BT major (12, 16, 17). The most prevalent reproductive complication in BT patients is iron induced hypogonadism, which is seen in 30 to 70% of BT patients worldwide (18–22). The endocrinopathy from transfusional iron overload appears to be more common in BT than in SCD (15, 23). However, during the past decades early diagnosis and more optimal treatment have significantly improved the quality of life of BT patients and increased their chance of childbearing (12, 14).

Both BT and SCD are genetic diseases in which the defects are expressed in the hematopoietic bone marrow. Transplantation of hematopoietic stem cell (HSC) derived from a HLA-compatible bone marrow donor can be curative in young BT and SCD patients (8, 24–26). Before HSC transplantation recipients are exposed to alkylating gonadotoxic therapies, which are associated with a substantial risk of premature ovarian insufficiency (POI) (27). Fertility preservation with ovarian tissue cryopreservation (OTC) is certainly indicated before HSC treatment, but it is not clarified whether fertility preservation is also recommended in those patients who are not candidates for HSC. It is not known to what extend the diminished hypothalamic-pituitary-ovarian function has a long-term effect on the ovary and the primordial follicles.

The aim of the present study was to evaluate the number of ovarian follicles, the follicle morphology, and the protein expression of essential follicle and oocyte health markers in girls and adolescents with BT and SCD undergoing OTC.

## Materials and Methods

### Patients

A total of 14 girls and adolescents with BT and four with SCD aged 2.8–17.4 years (mean age ± SD 10.4 years ± 4.2) had one entire ovary removed by laparoscopy for fertility preservation by OTC before HSC. Clinical data is listed in **Table 1**. A total of 43 girls and adolescents below the age of 18 years (range: 0.6–17.9 years, mean age 11.8 years) undergoing OTC was included as a reference group. Patients with genetic diseases were excluded from the reference group. The reference group included Danish patients with the following diagnoses: 15 sacoma, eight lymphoma, nine others benign, and 11 others malignant. No patients enrolled nor girls in the reference group received gonadotoxic treatment before OTC. Immunohistochemical analyses were performed in ovarian cortex sections of several reference patients; stains from one presentative patient diagnosed with a brain tumor aged 14.1 years old were presented as control. Ovarian follicle densities for some of the patients in the reference group have been published previously (28–30). Patients were only included if an ovarian cortex biopsy was obtained for histology in connection with OTC. All included patients were retrospectively included and underwent OTC between 2002 and 2019. Subjects 2, 3, and 17 underwent OTC in Melbourne. Subject 7, underwent OTC at Leeds University, UK, at the age of 9 years and was transplanted in Denmark at the age of 23 years (31). The remaining subjects underwent OTC at Laboratory of Reproductive Biology, Rigshospitalet, Denmark (**Table 1**).

**Table 1:** Clinical data on *β*-thalassemia (BT) and sickle cell disease (SCD) patients.

Subject no.	OTC center	Diagnose	Age at cryo (y)	Ovarian weight (g)	Ovarian tissue transplanted	Primordial follicles per mm^3^ in ovarian cortex
1	Copenhagen	BT	2.8	0.5	No	1,578
2	Melbourne	BT	6.0	0.4	No	210
3	Melbourne	BT	6.0	0.4	No	907
4	Copenhagen	BT	6.5	2.0	No	1,621
5	Copenhagen	BT	6.8	1.8	No	156
6	Copenhagen	BT	9.0	2.0	Yes (22 y)	124
7	Leeds	BT	9.0	NA	Yes (23 y)	1,449
8	Copenhagen	BT	9.1	0.8	No	566
9	Copenhagen	BT	12.0	1.1	No	136
10	Copenhagen	BT	12.0	3.5	No	273
11	Copenhagen	BT	13.6	2.8	No	161
12	Copenhagen	BT	15.4	6.9	No	22
13	Copenhagen	BT	16.7	1.7	No	41
14	Copenhagen	BT	17.4	9.2	No	215
15	Copenhagen	SCD	9.0	1.8	No	121
16	Copenhagen	SCD	9.3	2.0	No	402
17	Melbourne	SCD	10.3	0.6	No	277
18	Copenhagen	SCD	16.0	7.1	No	111

### Cryopreservation of Ovarian Tissue

The ovarian cortex was isolated and cut into small pieces as previously described for slow-freezing (32–34) and stored in liquid nitrogen. Additionally, one randomly chosen small piece of cortex was obtained for histological examination. The OTC schemes and collection of patient data were approved by the Ministry of Health (J. no. 30-1372) and by the Danish authorities to comply with the European Union tissue directive. All patients, or parents on behalf of their under-aged daughters, gave informed consent in writing.

### Histological Processing

Tissues were fixed in Bouin’s solution and embedded in paraffin. Tissues for density estimation were cut in 15 to 30 µm serial sections, de-paraffinated in xylene and stained with periodic-acid Schiff and counterstained with Mayer´s hematoxylin and erosin reagents (Sigma-Aldrich) for assessment of cell morphology. Sections of 5 µm were processed for immunohistochemical (IHC) staining.

### Follicle Density

Two methods were used to estimate the primordial follicle density in the ovarian cortex biopsies (5 × 5 × 2 mm^3^). In Copenhagen, the follicle density was estimated in 15 to 30 µm section using a mathematical model described by Schmidt and colleagues (35). In brief, this model was based on the fraction of sections, the mean primordial follicle diameter, and a correction factor (*α*) to account for the possibility of counting the same follicle more than once. Since the mean diameter of a primordial follicle is 44 µm (36) and the sections were 15 to 30 µm, there was a possibility to count the same follicle two or three times (35). All reference densities including the patient from Leeds were measured according to the method described by Schmidt (34). In Melbourne follicle density was measured in 5 to 6 µm sections by the method of McLaughlin and colleagues (37). In brief, all tissue sections were examined for the presence of follicles. To avoid overcounting, follicles were only assessed when the nucleolus was observed. The follicle density was determined by dividing the total number of follicles in the biopsy by the volume of tissue analyzed. To evaluate if the two methods of data collection were comparable a predictive model was used (37), which combine an age-related normative model for follicle population in the human ovary (38) and an age-related normative model for the volume of the human ovary (39). Comparison of data obtained by the two methods used shows good agreement using the predictive model (37).

### Immunohistochemical Staining

Ovarian tissue sections were de-paraffinated, rehydrated in ethanol followed by antigen retrieval in either 10 mM sodium citrate, pH 6 or 10 mM Tris, pH 9. Endogenous activity was inhibited using 1.5% peroxidase, nonspecific binding was inhibited using 1% bovine serum albumin (BSA) (Sigma Aldrich, Copenhagen, Denmark). Sections were incubated with primary antibodies overnight at 4°C, washed in phosphate-buffered saline with Tween20^®^ (PBST), and incubated with secondary horseradish peroxidase (HRP) conjugated antibody (Dako, Glostrup, Denmark) for 30 min at room temperature, visualized with DAB detection system (Abcam, cat.no.: ab64238), and counterstained with Meyer’s hematoxylin (Amplicon, Odense, Denmark). Details of antibodies and conditions are given in **Table 2**. Universal negative control serum (BioCare Medical) and antibody dilution buffer was used in place of primary antibody as negative controls and showed no staining (**Supplemental Figure 1**).

**Table 2:** Antibodies and conditions used for IHC analysis.

Antibody	Supplier	Cat. no.	Species	Concentration (µg/ml)
GDF9	Ansh Laboratories^a^	98/62A	Mouse	2.2
BMP15	Ansh Laboratories^a^	87/7A	Mouse	2.7
IGFBP4	Gift Claus Oxvig^b^	–	Mouse	5
PAPPA	Gift Claus Oxvig^b^	–	Mouse	1.0
STC2	Gift Claus Oxvig^b^	–	Mouse	2.5

a Texas, US.

b Department of Molecular Biology and Genetics—Molecular Intervention, Aarhus University, Aarhus, Denmark.

### Statistics

The GraphPad Prism 8.0.0 program (GraphPad Software, Inc., CA, USA) was used for statistical analysis. Linear regression was used to evaluate follicle density against age. Mann–Whitney U-test was used to evaluate if the BT and SCD follicle densities were different from the reference group. Statistical significance was defined as p-values <0.05.

## Results

### Girls With BT and SCD Have Normal Follicle Morphology and Density

**Figure 1:**
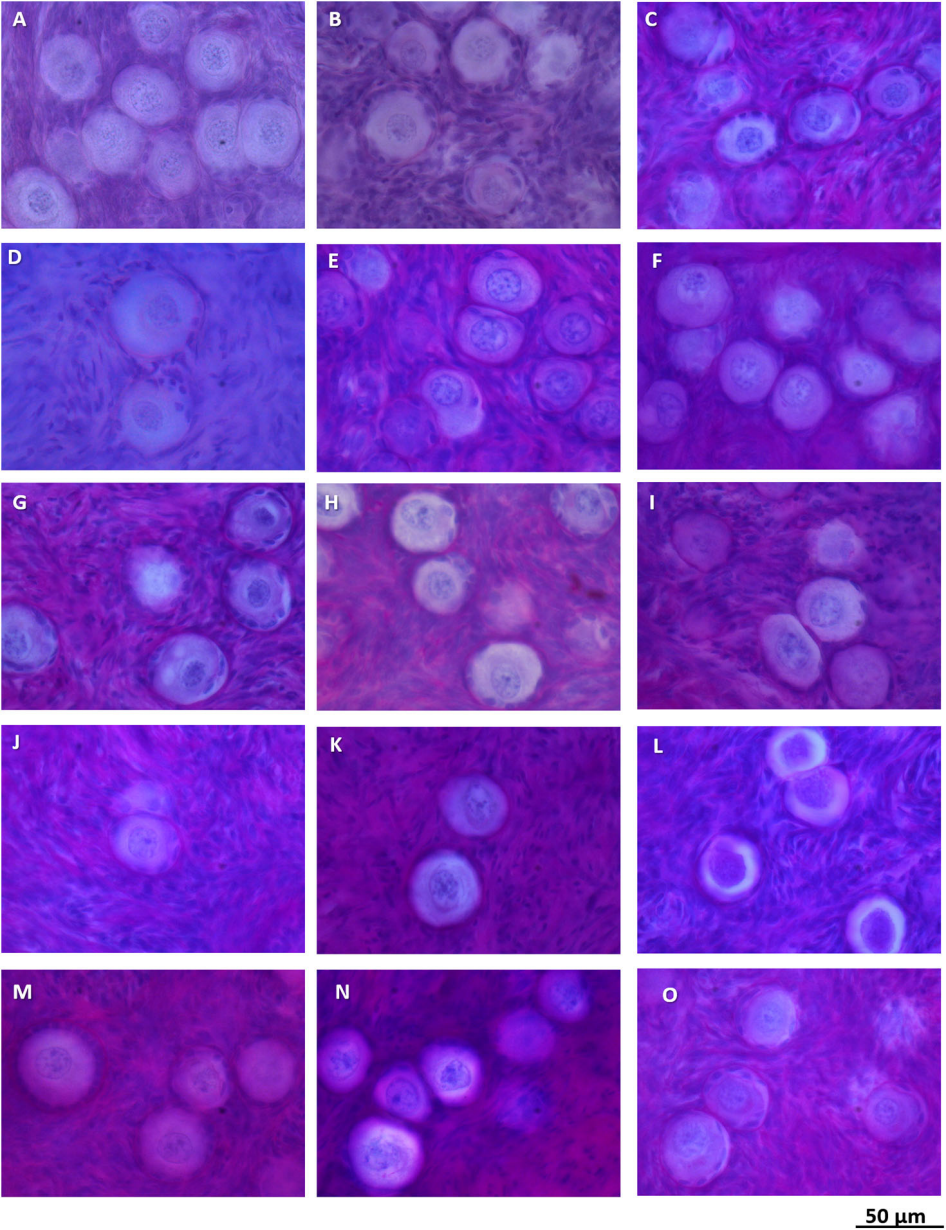
Normal follicle morphology in ovarian cortex in girls with *β*-thalassemia **(A–L)** and sickle cell disease **(M–O)**.

**Figure 2:**
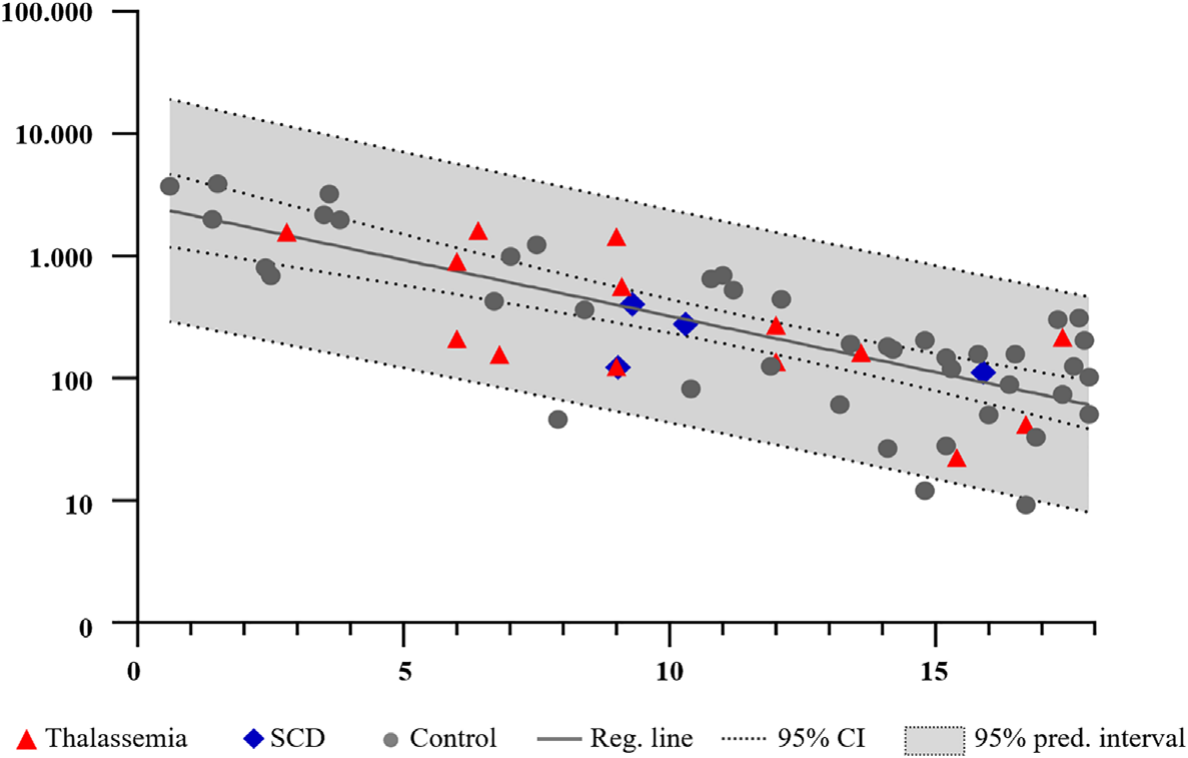
Follicle density (follicles/mm^3^) in normal ovaries (gray circles) and in girls and adolescents with thalassemia (red triangles) and sickle cell disease (SCD) (blue diamonds) (plotted against age. Soled line indicates the predicted follicle density; dotted line indicates the 95% confidence intervals (CI), and the gray area the 95% prediction interval.

In all cortical biopsies from BT and SCD the follicles appeared morphologically normal. Most of the primordial follicles presented with a centrally located primordial nucleus surrounded by a single layer of orderly flattened granulosa cells. Atretic follicles and abnormal follicles with irregular granulosa cell layers were also detected, which is common in children ovaries (40, 41); examples are presented in **Figure 1**. Follicle densities in BT and SCD ovaries were in all cases within the 95% prediction interval of the age-matched reference group, and the number of follicles did not differ significantly from the reference group (p = 0.71) (**Figure 2**). The exact follicle densities are presented in **Table 1**. A significant negative linear association between follicle density and age at OTC was found both in the BT and SCD group and in the reference group (p = 0.0016, p < 0.0001, respectively).

### Normal Protein Profiles in Primordial Follicles From BT and SCD Girls

**Figure 3:**
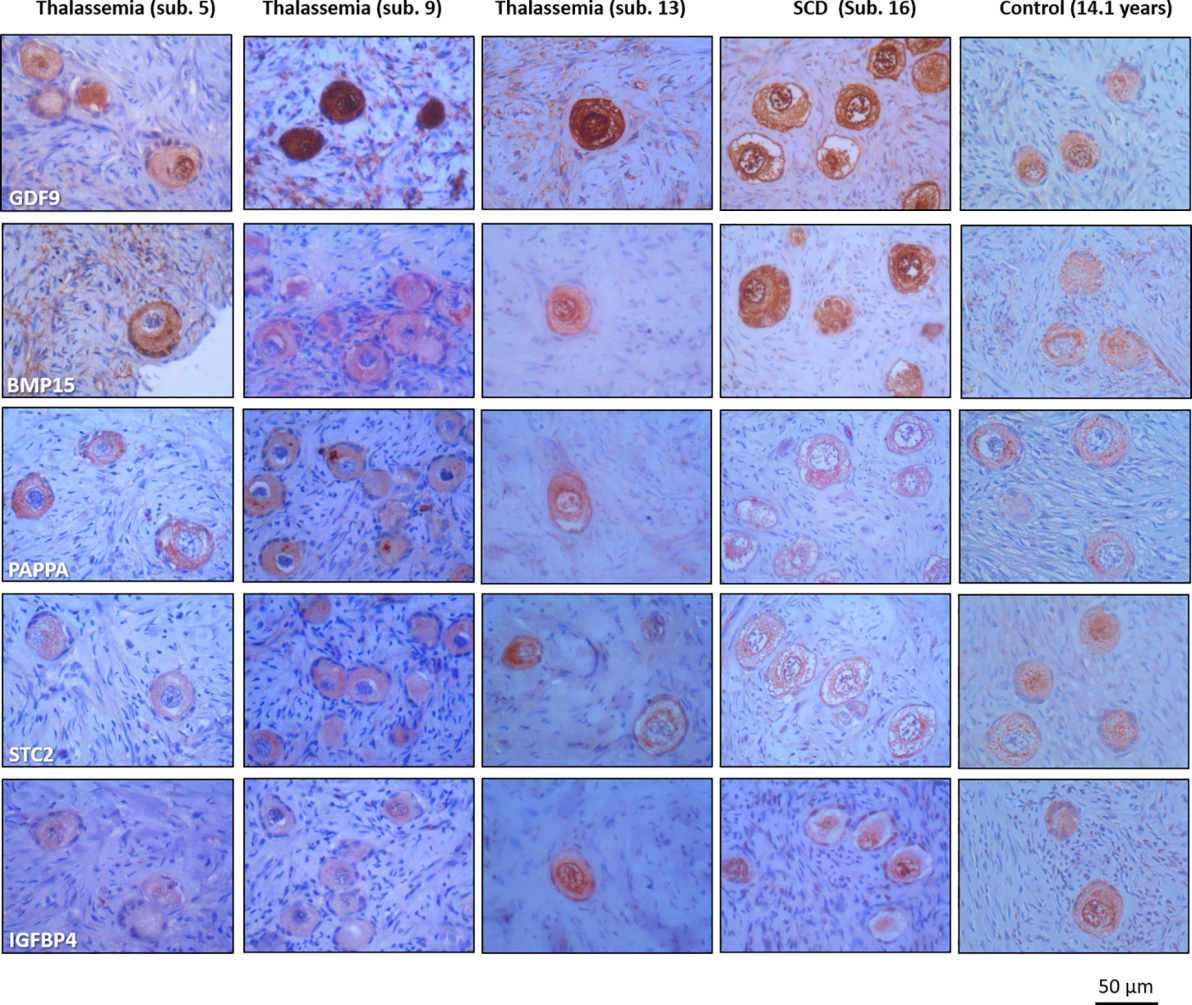
Similar detection of growth differentiation factor 9 (GDF9). Bone morphogenetic protein 15 (BMP15), pregnancy-associated plasma protein A (PAPPA), stanniocalcin 2 (STC2), and insulin-like growth factor-binding protein 4 (IGFBP4) in ovarian cortex with thalassemia and sickle cell disease and in one age-matched reference biopsy.

Immunohistochemical analysis (IHC) was used to detect the presence of two oocyte specific markers growth differentiation factor 9 (GDF9), bone morphogenetic protein 15 (BMP15) (42–44), and three members of the insulin-like growth factor (IGF) system including pregnancy-associated plasma protein A (PAPPA), stanniocalcin 2 (STC2), and insulin-like growth factor-binding protein 4 (IGFBP4), which are known to be expressed by developing follicles (45–50). IHC was used in three BT and one SCD ovarian biopsies (subjects 5, 9, 13 and 16, respectively) and in additional subjects from reference group. Staining from one 14.1-year-old illustrative subject from the reference group, diagnosed with a brain tumor, was presented (**Figure 3**). GDF9 and BMP15 located primarily to the oocyte, a weak staining was seen in the stroma. PAPPA, STC2 and IGFBP4 located to primordial follicles, weak staining are seen in the stoma supporting previous reports (45, 46, 49, 50) (**Figure 3**).

### Transplantation of Ovarian Cortex Frozen Before Puberty Can Give Rise to Livebirth

Two patients with BT (subjects 6 and 7), both aged 9 years at OTC had five ovarian cortex pieces (out of a total of eight pieces frozen) transplanted in Denmark at the age of 22 and 23 years (**Table 1**). Both patients were menopausal at transplantation and in both cases serum hormone levels went back to normal three to four months after transplantation. Within a year after transplantation, subject 7 conceived following IVF treatment where the oocytes originated from the transplanted tissue and delivered a healthy baby (31). Subject 6 no longer has a pregnancy wish (data not published).

## Discussion

The present study characterized ovarian follicles in girls and adolescents with BT and SCD and explored the feasibility of fertility preservation by OTC in these patients. Follicle density, morphology, and the expression of essential follicle- and oocyte specific proteins in early stage follicles were comparable to an age-matched reference group. In all cases the estimated numbers of primordial ovarian follicles were within the 95% prediction interval of normal age-matched reference ovaries, illustrating that the number of follicles is not compromised by these diseases during childhood and puberty. BT and SCD patients originated from Denmark (11 BT, 3 SCD), Australia (2 BT, 1 SCD), and UK (1 BT), whereas all reference ovaries were Danish. It cannot be excluded that the evaluated parameters would have been different in an Australian/UK reference cohort, although the methods used were cross-validated and no differences were found (37). In all case and reference patients, most of the follicles appeared morphologically normal. A small fraction of abnormal follicles was detected in all subject, which is normal in young and immature ovaries.

In four cases, there were ovarian material available for IHC analyses, and the expression of three proteins involved in the regulation of folliculogenesis (PAPPA, STC2, IGFBP4) (49) and oocyte health (GDF9 and BMP15) (42–44) were evaluated. All markers were expressed in both BT, SCD and reference tissues. This indicates that primordial follicles from BT and SCD patients appear to be normal with respect to these essential proteins. In contrast, iron overload has previously been detected in follicle fluids from BT patients and it has been suggested to induce oxidative stress that compromises follicle health (51). The presented data is based on a limited number of BT and SCD patients and it cannot be excluded that the findings does not reflect the general population with BT and SCD. Moreover, the present study cannot exclude that high iron concentration may have negative impact on growing follicles, though it appears not to be the case in primordial follicles.

Taken together, these findings suggest that the pool of primordial follicles, is normal. This argue in favor of performing OTC, which preserves the primordial follicles only. OTC is indeed indicated before HSC treatment which carries a significant risk of subsequent infertility. When the tissue is transplanted after HSC the iron level and the endocrine milieu will be normal and normal follicular growth will be expected. OTC may also be considered in girls who are not facing HSC transplantation, since it is not clarified whether a prolonged systemic high iron level will compromise follicle health on the long run.

Two patients in the cohort had tissue transplanted (subjects 6 and 7). Both cases had tissue frozen before puberty at the age of 9 years. These patients were 22 and 23 years of age and menopausal at transplantation; in both cases serum hormone levels went back to normal three to four months after transplantation, illustrating the functionality of the cryopreserved pre-pubertal ovarian tissue. Subject 7 became pregnant following IVF treatment within a year from transplantation and gave birth to a healthy boy, being the first proof of concept of truly pre-pubertal ovarian tissue giving rise to healthy offspring (31).

Globally, successful OTCs have been reported in three BT cases and six SCD cases (31, 52–58). Moreover, of these patients three with BT and three with SCD have been transplanted, regained ovarian function, and pregnancies with healthy live-births have been reported in all three TB cases and in two of the SCD cases (31, 52, 53, 55, 56). Hereby, illustrating that OTC can regain ovarian function in these patient groups. A total of 15 oocyte/embryo cryopreservations have been reported in SCD, with no reported transfers so far (9, 57, 59, 60), making it difficult to evaluate this fertility preserving strategy. Women with SCD undergoing ovarian stimulation prior to oocyte and embryo cryopreservation have an increased risk of ovarian hyperstimulation syndrome (OHHS), thrombosis, vaso-occlusive events, and painful crises (9, 57, 59, 60), all complications which may favor OTC. However, SCD patients have an increased risk of severe pulmonary complications during general anesthesia, which can lead to multi-organ failure and death (61, 62), which has to be considered before deciding on which fertility preserving strategy to use.

The present study found no differences in the density of primordial follicles, morphology, and expression of follicle- and oocyte specific proteins in girls with BT and SCD compared to the age-matched reference group, suggesting that primordial follicles may not be affected by iron overload. These findings together with the reported cases of successful ovarian tissue transplantations giving rise to life-births in women with BT and SCD, suggest that OTC should be offered to girls and adolescent with BT and SCD, before HSC. OTC may also be considered in young BT and SCD patients, who are not facing and HSC transplantation since it is not clarified whether a prolonged systemic high iron level will compromise follicle health and fertility in the long run.

## Data Availability Statement

The raw data supporting the conclusions of this article will be made available by the authors, without undue reservation, to any qualified researcher on request.

## Ethics Statement

This study was approved by the Ministry of Health (J. no. 30-1372) and by the Danish authorities to comply with the European Union tissue directive. Written informed consent to participate was provided by the parrents /participants’ legal guardian.

## Author Contributions

LM designed the project, wrote the paper, cryopreserved ovarian tissue, measured follicle density in the Danish cohort, did IHC staining, and analyzed the data. CA, SK, SP, and DG cryopreserved ovarian tissue. DG measured the follicle density in the Australian cohort. JB assisted in statistical analysis. EE and KM recruited patients and did the ovariectomies. AK and BK designed antibodies (GDF9 and BMP15), contributed to design and interpretation of the data. CA designed the project and wrote the paper. All authors contributed to the article and approved the submitted version.

## Funding

The study was designed, conducted, analyzed, and reported entirely by the authors. This paper presents independent research funded by grants from Rigshospitalets Forskningspuljer, the International EU project ReproUnion.

## Conflict of Interest

AK and BK were employed by the company Ansh Labs.

The remaining authors declare that the research was conducted in the absence of any commercial or financial relationships that could be construed as a potential conflict of interest.
