# Supplementary figures and images for: Consequences of β-Thalassemia or Sickle Cell Disease for Ovarian Follicle Number and Morphology in Girls Who Had Ovarian Tissue Cryopreserved
Source: Front Endocrinol (Lausanne). 2021 Jan 15;11:593718. doi: 10.3389/fendo.2020.593718 (PMC7844814; doi:10.3389/fendo.2020.593718)

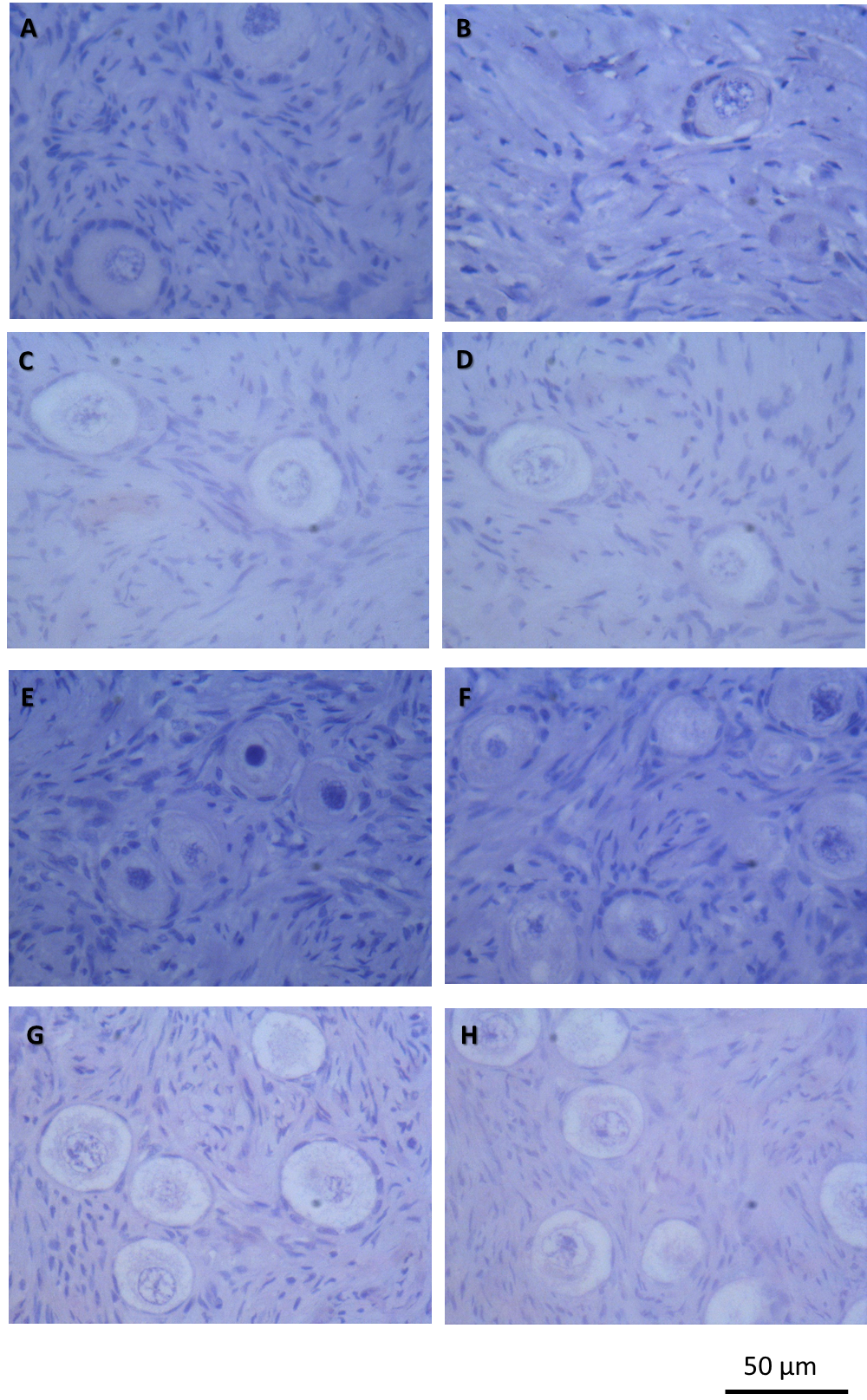

Supplement: Supplementary file 1 [file Image_1.tif]
